# Animal welfare requirements in publishing guidelines

**DOI:** 10.1177/00236772221097825

**Published:** 2022-06-21

**Authors:** Amanda L Novak, Darren J. Shaw, R. Eddie Clutton

**Affiliations:** 1 Bioresearch and Veterinary Services, The University of Edinburgh, UK; 2 Royal (Dick) School of Veterinary Studies, The University of Edinburgh, UK; 3 Wellcome Trust Critical Care Laboratory for Large Animals, The Roslin Institute, UK

**Keywords:** animal welfare, ethics, 3Rs, ARRIVE, journal publishing guidelines

## Abstract

Descriptions of measures taken to optimize animal welfare are often absent from scientific reports of animal experiments. One reason may be that journal guidelines inadequately compel authors to provide such information. In this study, online English language versions of the ‘Guidelines to authors’ (GTAs) from 54 national biomedical journals were examined for neutral (unrelated to welfare) and non-neutral keywords referring to: animal welfare; the ‘3Rs’; the ARRIVE (2010) guidelines, and regulations pertaining to animal experimentation. Journals were selected from nine countries (UK, US, China, Canada, India, Brazil, Germany, Japan and Australia) and seven biomedical specialties (oncology, rheumatology, surgery, pharmacology, medicine, anaesthesia and veterinary medicine). Total GTA word counts varied from 1137 to 31,609. The keyword count identified per category were expressed per myriad (10,000) of total word count. One-way analyses of variance followed by post hoc Tukey pairwise comparisons revealed greater non-neutral per myriad word counts for (a) veterinary GTAs compared with medicine, oncology, rheumatology or surgery; (b) British, compared with Australian, Canadian, German and Japanese GTAs; and (c) no differences between non-neutral categories. The English language versions of GTAs of British and veterinary medical journals contain more words associated with animal welfare, the 3Rs and the ARRIVE guidelines than those from eight other countries and six other medical specialities. The exclusion of ‘national’ language versions from analysis precludes attempts to identify national differences in attitudes to laboratory animal welfare.

## Exigences de bien-être des animaux dans les lignes directrices de publication

Les descriptions des mesures prises pour optimiser le bien-être des animaux sont souvent absentes des rapports scientifiques d'expériences animales. Cela peut être dû au fait que les lignes directrices de la revue n’obligent pas de manière inadéquate les auteurs à fournir de telles informations. Dans cette étude, les versions en ligne en anglais des « lignes directrices destinées aux auteurs » (LDA) de 54 revues biomédicales nationales ont été examinées en fonction de mots-clés neutres (sans rapport avec le bien-être) et non neutres se référant au bien-être animal, au principe des « 3R », ainsi qu’aux lignes directrices et règlements ARRIVE (2010) relatifs à l'expérimentation animale. Les revues ont été sélectionnés parmi: 9 pays (Royaume-Uni, États-Unis, Chine, Canada, Inde, Brésil, Allemagne, Japon et Australie) et 7 spécialités biomédicales (oncologie, rhumatologie, chirurgie, pharmacologie, médecine, anesthésie et médecine vétérinaire). Le nombre total de mots des LDA variait de 1 137 à 31 609. Le nombre de mots-clés identifiés par catégorie a été exprimé *par myriade* (10 000) de nombre total de mots. Des analyses unidirectionnelles de la variance suivies de comparaisons post-hoc de Tukey par paires ont révélé des nombres non neutres par myriade de mots plus importants pour: a) les LDA vétérinaires par rapport à la médecine, à l’oncologie, à la rhumatologie ou à la chirurgie, et b) les LDA britanniques, par rapport à celles d’Australie, du Canada, d’Allemagne et du Japon; c) aucune différence entre les catégories non neutres. Les versions en anglais des LDA des revues médicales britanniques et vétérinaires contiennent plus de mots associés au bien-être des animaux, aux 3R et aux lignes directrices ARRIVE que celles de huit autres pays et de six autres spécialités médicales. L’exclusion des versions linguistiques « nationales » de l’analyse exclut les tentatives d’identification des différences nationales d’attitudes à l’égard du bien-être des animaux de laboratoire.

## Tierschutzbestimmungen in Publikationsrichtlinien

In wissenschaftlichen Berichten über Tierversuche fehlen häufig Einzelheiten zu den Maßnahmen, die für optimalen Tierschutz getroffen wurden. Dies kann u. U. daran liegen, dass die Leitlinien der Publikationen die Autoren nur unzureichend dazu verpflichten, solche Informationen bereitzustellen. In der vorliegenden Studie wurden englischsprachige Online-Versionen der „Guidelines to authors “(GTA) von 54 nationalen biomedizinischen Zeitschriften auf neutrale (ohne Bezug zum Tierschutz) und nicht-neutrale Schlüsselwörter untersucht, die sich auf den Tierschutz, die „3R“, die ARRIVE-Leitlinien (2010) und auf Vorschriften zu Tierversuchen beziehen. Es wurden Zeitschriften aus 9 Ländern (Vereinigtes Königreich, USA, China, Kanada, Indien, Brasilien, Deutschland, Japan und Australien) und 7 biomedizinischen Fachgebieten (Onkologie, Rheumatologie, Chirurgie, Pharmakologie, Medizin, Anästhesie und Veterinärmedizin) ausgewählt. Die Gesamtwortzahl der GTA lag zwischen 1.137 und 31.609. Die Anzahl der pro Kategorie ermittelten Schlüsselwörter wurde in *Myriade* (10.000) der Gesamtwortzahl ausgedrückt. Einweg-Varianzanalysen, gefolgt von paarweisen Post-hoc-Tukey-Vergleichen, ergaben eine höhere Anzahl nicht-neutraler Wörter pro Myriade für: a) veterinärmedizinische GTA im Vergleich zu Medizin, Onkologie, Rheumatologie oder Chirurgie und b) britische GTA im Vergleich zu australischen, kanadischen, deutschen und japanischen GTA; c) keine Unterschiede zwischen nicht-neutralen Kategorien. Die englischsprachigen Versionen von GTA britischer und veterinärmedizinischer Zeitschriften enthalten mehr mit Tierschutz, den 3R und den ARRIVE-Leitlinien verbundene Wörter als die aus den acht anderen Ländern und den sechs anderen medizinischen Fachgebieten. Der Ausschluss der „nationalen “Sprachversionen von der Analyse steht dem Versuch entgegen, nationale Unterschiede in der Einstellung zum Schutz von Versuchstieren zu ermitteln.

## Requisitos para el bienestar animal que deben incluirse en las directrices sobre política editorial

Las descripciones de las medidas adoptadas para optimizar el bienestar de los animales no suelen aparecer en los informes científicos sobre experimentos con animales. Uno de los motivos podría ser que las directrices de las revistas no obligan suficientemente a los autores a proporcionar esta información. En este estudio, se examinaron las versiones en línea en inglés de las “Directrices para autores” de 54 revistas biomédicas nacionales buscando palabras clave neutrales (no relacionadas con el bienestar) y no neutrales que hicieran referencia a: el bienestar animal; las “3R”; las directrices ARRIVE (2010) y las regulaciones relativas a la experimentación con animales. Se seleccionaron revistas de: 9 países (Reino Unido, Estados Unidos, China, Canadá, India, Brasil, Alemania, Japón y Australia) y 7 especialidades biomédicas (oncología, reumatología, cirugía, farmacología, medicina, anestesia y veterinaria). El recuento total de palabras de las Directrices para autores varió de 1137 a 31 609. El recuento de palabras clave identificadas por categoría se expresó *por miríada* (10 000) del recuento total de palabras. Los análisis de varianza de una vía, seguidos de comparaciones post hoc de Tukey por pares, revelaron un mayor recuento de palabras no neutrales por miríada para: a) las Directrices para autores de veterinaria en comparación con las de medicina, oncología, reumatología o cirugía, y b) las Directrices para autores británicas en comparación con las australianas, canadienses, alemanas y japonesas; c) ninguna diferencia entre las categorías no neutrales. Las versiones en inglés de las Directrices para autores de las revistas médicas británicas y veterinarias contienen más palabras asociadas al bienestar animal, las 3R y las directrices ARRIVE que las de otros ocho países y otras seis especialidades médicas. La exclusión del análisis de las versiones en idiomas “nacionales” impide intentar identificar las diferencias nacionales en las actitudes respecto al bienestar de los animales de laboratorio.

## Introduction

In seeking to increase experimental reproducibility, the ARRIVE (Animal Research: Reporting of In Vivo Experiments) guidelines attempted to improve reporting standards in animal experiments.^1^ Among other things, ARRIVE recommended the inclusion of details relating to animal welfare, as well as the implications of experimental findings for the replacement, refinement or reduction (the 3Rs) of the use of research animals. Unfortunately, ARRIVE (2010) has failed to achieve this, prompting (a) Carbone to conclude that the scientific literature could not be trusted to present full details on procedures that have critical effects on animal welfare,^2^ and (b) the development and launch of ARRIVE 2.0.^3^ This has been supported by studies quantifying the description of analgesic use in several laboratory animal species,^4–8^ and the abuse of neuromuscular blocking agents in pigs.^9^ Leung and co-workers asserted that ARRIVE had not improved the reporting quality of papers in animal welfare, analgesia or anaesthesia.^10^

One reason for low compliance may have been low awareness amongst international scientists—despite the availability of the ARRIVE guidelines in Portuguese, Brazilian Portuguese, Spanish, Chinese, Italian (2014), Japanese (2016), French and Korean (2017). While Italian language versions existed in 2016, a subsequent questionnaire-based study canvassing Swiss-registered in vivo researchers (*n* = 1891) found more than 51% of respondents claimed they had never heard of the ARRIVE guidelines.^11^ Studies of national ARRIVE uptake have focussed on individual countries, e.g. China and Korea,^12,13^ and so comparing ‘national’ effects are difficult. That said, in testing the requested use of an ARRIVE checklist on article submission, Hair and colleagues found the country of origin of corresponding authors, specifically the United States (US), Brazil, China, Germany, Canada, Japan and 47 others, had no effect on overall compliance.^14^

That animal welfare-related subjects are under-reported in scientific publications is surprising given that, in most countries, training in laboratory animal ethics, welfare, the law and associated topics is a mandatory prerequisite to the licensing of animal experiments and participating personnel. Such courses usually emphasise the importance of the 3Rs principle because these provide an ethical defence for using animals in research. More pragmatically, many funding bodies, legislative authorities, AWERBs (Animal Welfare and Ethical Review Bodies) or IACUCs (Institutional Animal Care and Use Committee) require evidence of commitment to the 3Rs principle before studies are funded and/or approved. That details of animal welfare and/or ethics are frequently absent from scientific reports raises the possibility that the teaching objectives of these training courses are not being met. Alternatively, such details are possibly considered unnecessary by both authors and the editorial process alike. This then indicates a possible ignorance of the effects of details such as pain management on data quality, the validity of subsequent conclusions and experimental replicability.^15,16^ It may also reflect an ignorance of the refinement principle, elements of which, e.g., anaesthetics, are a societally expected and legally regulated condition of the use of animals in research—at least in the United Kingdom (UK).^17^

Han and colleagues found that completing a checklist at manuscript submission was associated with improved reporting of key methodological information in preclinical animal studies. ^18^ Similarly, when the Nature Publishing Group introduced a bespoke mandatory reporting checklist, there was an improvement in the reporting of bias risks to a previously unattained level.^19^ Additionally, Carlijn and co-workers interviewed experts in the field of Anaesthesiology, Pharmacology, Oncology, Rheumatology and Laboratory animal science and found that authors would adopt a ‘Publication Checklist to Improve the Quality of Animal Studies’ if journals required adherence.^20^ These findings indicate the potential for editorial mandates to improve the reporting of welfare details. This already exists insofar as most publishing houses and the Committee on Publication Ethics (COPE) request—as a minimal requirement—that AWERB (or IACUC) approval be recorded in submitted manuscripts. This goes some way to ensuring that journals do not publish unethical or unlawful studies irrespective of major differences in international laboratory animal legislation.^21^

One explanation for inadequate animal welfare details in scientific articles is that their inclusion is ineffectually mandated in the journal’s ‘guidelines to authors’ (GTAs), which for many authors is the final checklist before article submission. Indeed, for those scientists who are not required to undergo ethics and welfare training, and who are unaware of the ARRIVE guidelines, GTAs may be the only source of information that prompts them to describe the ethical and welfare implications of their work. Previous workers have examined GTAs for references to ARRIVE 2010 and found few, if any such references.^12,22^ Sims looked only at emergency medicine journals and found that 40.7% of ‘Instructions for Authors’ failed to reference a single reporting guideline,^22^ whereas Zhang reviewed only journals from mainland China and found none of the GTAs of 238 *in vivo* journals referred to the ARRIVE guidelines.^12^ The current manuscript reports the first study, to our knowledge, to look for more diverse evidence of laboratory animal welfare concerns.

This study aimed to examine and quantify the emphasis placed on animal welfare, the 3Rs, ARRIVE guideline compliance and the levels of regulatory approval in the GTAs of journals from the nine countries reportedly accounting for the greatest laboratory animal use, i.e., the UK, the US, China, Canada, India, Brazil, Germany, Japan and Australia, and in the flagship journals of seven medical specialties associated with animal experimentation, i.e., oncology, rheumatology, surgery, pharmacology, medicine, anaesthesia and veterinary medicine. A secondary objective was to determine whether national and/or speciality differences existed in the emphasis—if any—placed on each of these four categories.

## Materials and methods

English-language versions of the GTAs of 54 selected journals were obtained from the official journal websites. They were examined for selected keywords which (a) confirmed the journal published animal experiments; (b) reflected the emphasis the journal placed on animal welfare; (c) showed the journal's promotion of the ARRIVE guidelines, or the principles of those guidelines; (d) indicated the extent to which the journal endorsed the 3Rs principle; and (e) revealed the level of authority approving the experiment.

Journals were selected on the basis of (a) their primary national origin; and (b) the medical speciality for which they catered. Journals from the US, China, the UK, Germany, Japan, Canada, India, Brazil and Australia were selected because (a) they were reported as being amongst the world's 12 greatest users of laboratory animals,^23^ and/or (b) they had been selected in previous studies examining ARRIVE compliance.^12,14^ Five of the seven medical specialities represented in this study (pharmacology, oncology, surgery, rheumatology and internal medicine) were selected on the basis of their (a) reliance on animal studies; (b) potential association with noxious procedures, and (c) similarity to a range of specialities examined previously for similar purposes.^5,20,22^ Anaesthesia journals were examined because this subject is a major element of experimental refinement,^4,24^ whereas veterinary medical journals were reviewed in the belief that they would be more aware of *in vivo* reporting guidelines and prioritize animal welfare, and so represent exemplars.^25^

Journals were found by searching ‘country’ and ‘specialty’ in an online directory of open access journals (DOAJ) and Google search using Google Chrome (installation version 54.0.2840.87 m). Journals within each speciality were selected on the basis that they represented the organ of that speciality’s principal professional body for each of the countries examined. Only journals whose guidelines were available online and in English were analysed. In all but a single case, there was only one journal that met all inclusion criteria per category. It was later clarified when reviewing other guidance on *The Journal of Clinical Pharmacology* website that *in vitro* research would not be consider for publication. Therefore, *The Journal of Pharmacology and Experimental Therapeutics* was selected.

The selection and categorization of keywords was an iterative process that began by finding the word ‘animal(s)’ in the GTAs and examining adjacent text for associated words that could be categorised as (a) neutral, i.e. terms linked with animal use but without welfare connotations; (b) welfare-related; (c) 3Rs-related; (d) ARRIVE- related (or achieving ARRIVE objectives); and (e) pertaining to regulation, approval, authority and/or control (regulatory). As more guidelines were analysed, keywords were re-categorised, excluded or added—a process that necessitated re-examining those guidelines that had already been processed. Keywords qualifying for inclusion in both ARRIVE and other categories were allocated to the former, on the basis that their presence in the GTA was taken to indicate compliance (knowing or otherwise) with the ARRIVE objectives. Revision continued until no further changes to the keyword list were required and had been applied to all selected guidelines. A rigorous (and repeated) analysis of the context of putative keywords ensured that only those linked directly to animals were counted. Thus, the keyword ‘pain’ was not counted if it related to the human experience.

The first stage of analysis involved converting online guidelines into a Microsoft Windows .doc format. Corrupted text was then re-configured using MS Word; for example, fused expressions (e.g. IMinjection) or fragmented words (e.g. IM inj ection) were corrected. The word count was recorded using Microsoft Word’s word count function. Where ‘animal’ referred to experimental studies, the word was colour-highlighted according to category and the entire section examined for the keywords listed. The number of keywords counted under each category was recorded in an Excel table, summated and expressed as a proportion of the total word count on a *per myriad*, i.e. one in 10,000 (or 0/000) basis.

The GTAs were analysed by two (AN and EC) authors examining different countries; AN examined the US, China, Germany, Canada and Brazil. Guidelines were selected and analysed over a 5-month period (March–July 2020). Consistent scoring was promoted by conducting several cycles of keyword re-categorisation, contextual revision and re-application. After the final iteration, consistency was tested with both authors examining all Canadian speciality guidelines and comparing the word counts in each category. Adjustments were made as necessary based on consensus of the authors, and a final extraction manual was created to standardise the process.

One-way analyses of variance (ANOVA) were used to test for differences in the use of non-neutral words by country, journal type and terms of interest. Where overall differences were observed, post hoc Tukey pairwise comparisons were performed. A *P* value <0.05 was taken to indicate statistical significance. All analyses were carried out in R v.4.1.0 (2021, The R Foundation for Statistical Computing), with the packages *multcomp* (v.1.4-17) and *multcompview* (v.0.1-8) used for the post hoc Tukey pairwise comparisons.^26,27^

### Animals

This study did not involve the use of animals directly.

## Results

Of the calculated maximum (9 × 7) of 63 journal guidelines, only 61 were identified: Australian journals devoted to rheumatology and pharmacology do not exist. Of the 61 available, only 54 met selection criteria because 7 journals either did not (a) publish animal research; (b) publish GTAs; (c) mention ‘animal’ in their guidelines; (d) publish guidelines in English; or (e) were international or regional rather than national (Table 1). *The Journal of Pharmacology and Experimental Therapeutics* was selected over *The Journal of Clinical Pharmacology* to represent ‘pharmacology’ in the US because the former described itself as a ‘research journal’. Two German journals (representing veterinary medicine and anaesthesia) were not analysed because their guidelines were unavailable in English.

**Table 1.** Abbreviated (Index Medicus; https://woodward.library.ubc.ca/research-help/journal-abbreviations/) titles of 61 national ‘flagship’ journals representing seven medical specialities and publishing animal experiments in English. Oblique strokes indicate journal unavailability in that subject area/country. Shaded cells indicate journals failing to meet all inclusion criteria (see text for details). The word count of the corresponding GTAs of selected journals is shown in parentheses.

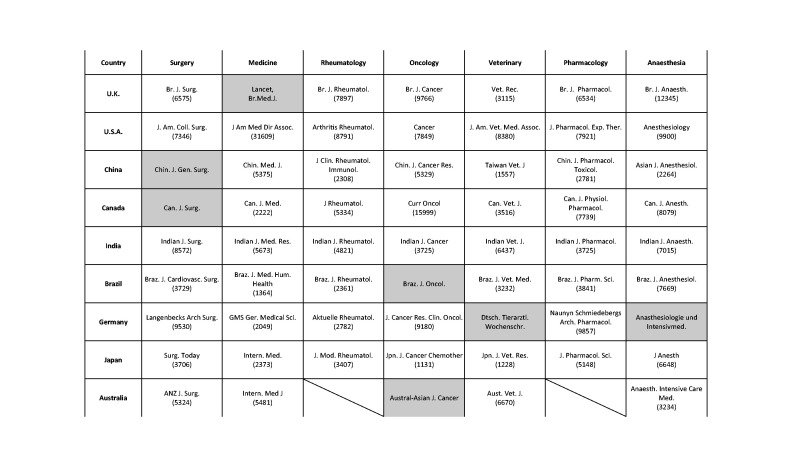

After seven cycles of identification and re-categorisation, a total of 73 keywords were identified and divided into neutral (*n* = 18), welfare-related (*n* = 13), 3R’s (*n* = 12) and ARRIVE (*n* = 28) categories (see Table 2; Categories). Five levels of regulation were identified and ranked according to the level of authority (Table 2; Regulatory). There was strong agreement between the two authors carrying out keyword identification and categorisation when they independently analysed the six available Canadian guidelines. Both recognized the same 4 keywords for the medical and rheumatology guidelines, the same 5 in oncology, and the same 10, 11 and 13 keywords in the veterinary, anaesthesia and pharmacology guidelines, respectively. Differences in categorisation occurred at a rate of 5 words per 100.

**Table 2.** Categories: Keywords used to indicate concerns with animal use (neutral), animal welfare (welfare), the 3Rs principle, awareness of the ARRIVE guidelines or its principles (ARRIVE) and the level of regulatory observance required (regulatory). ^A^*word* and *word*^A^ indicates the word ‘animal’ precedes, or follows a keyword, respectively. IACUC, Institutional Animal Care and Use Committee; 3Rs, Replacement, Reduction and Refinement; NC3Rs, The National Centre for the Replacement, Refinement and Reduction of Animals in Research; ILAR, Institute for Laboratory Animal Research. Regulatory: Ranked levels (1 = lowest) of examples of regulatory, advisory and/or approving bodies identified in authors guidelines.

Categories				Regulatory	
Neutral	Welfare	3Rs	ARRIVE	Level	Example
*animal* ^A^ *use* ^A^ *study* ^A^ *research* ^A^ *experiment* ^A^ *trial* ^A^ *tissue(s)* ^A^ *preparation* ^A^ *cells* ^A^ *material* ^A^ *method(s) * ^A^ *model(s) * ^A^ *science* ^A^ *species* ^A^ *subject(s) * ^A^ *hostlaboratory* ^A^ *transgenic^A^client-owned* ^A^	*welfarehealthpainsufferingstressdistressanalgesiaanaesthesiaadverseeuthanasiakilldestroycare*(but not linked with IACUC)	*3Rs*^A^*free*^A^*alternatives*^A^*replacement^A^reduction*^A^*ethicshumanerefinementhumane end-pointharm:benefit ratioNC3RsILAR*(or other 3R's related bodies)	*ARRIVEARRIVE HTMLcharacteristics speciesstrainsexagespeciesmodel justification experimental methodallocationrandomisation sourcesupplyhousingbeddingcagegroupinghusbandrylighttemperaturehumiditygroup cohortnumbershealth statusinclusionexclusion model limitations3Rs implications*‘unspecified’ reporting guidelines	1) Local institutional review	Animal Welfare and Ethical Review Bodies (*AWERB*)
					Institutional Animal Care and Use Committee (*IACUC*)
					Australia: Animal Care and Ethics Committee (*ACEC*)
					Brazil: Ethics Committee on the Use of Animals (*CEUA*)
				2) National guidelines	The Canadian Council on Animal Care standards (*CCAC*)
				3) National funding body guidelines	CCAC Certificate of GAP-Good Animal Practice, US Public Health Services (*PHS*) Act requires consistency with the Guide for the Care and Use of Laboratory Animals.
				4) National legislation	UK: Animal (Scientific Procedures) Act 1986 *A(SP)A 1986*
					USA: Animal Welfare Act (*AWA*),
					Health Research Extension Act (1985).
					EU: transcribes EU Directive 2010/63/EU into national law
				5) Multi-national legislation	European Union's Directive 2010/63/EU

**Figure 1.**
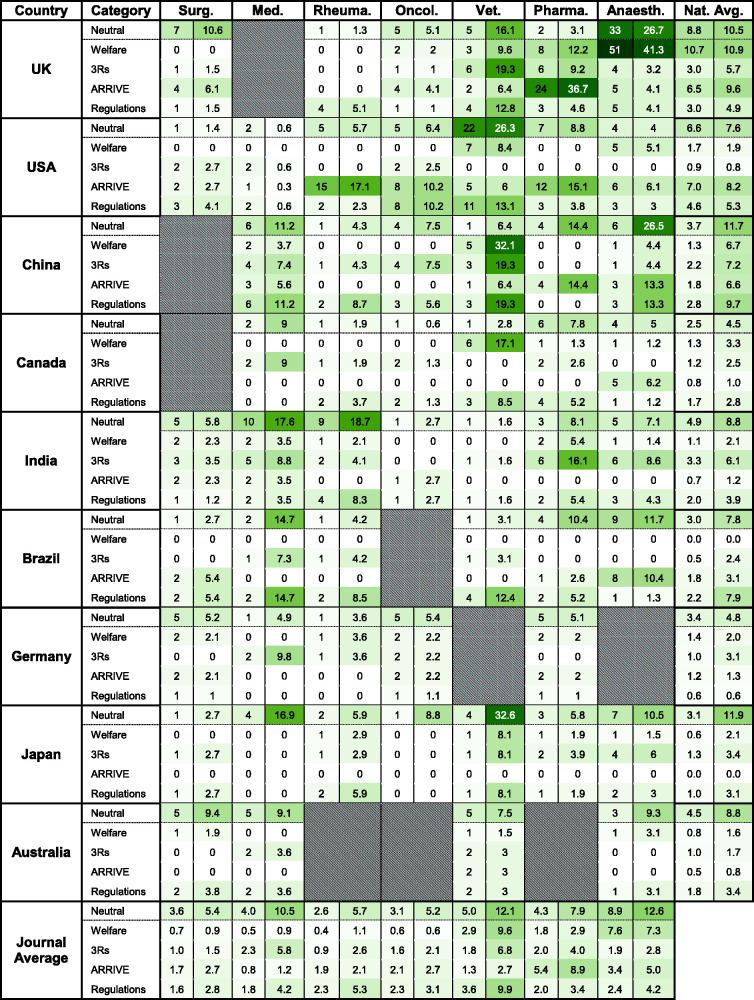
A ‘heat map’ of neutral and non-neutral (welfare-, 3Rs- and ARRIVE-related) keyword numbers in ‘Guidelines to authors’ (GTAs) from seven speciality medical journals from nine countries. The left-hand column under each journal category is the absolute word count. The right-hand column is the keyword count per myriad of total words. Grey hatched cells indicate national speciality journal guidelines that did not exist or failed to meet inclusion criteria. The word count values and corresponding cell colours are:
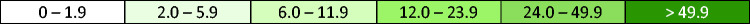

There was a 25-fold difference in the total word counts between the briefest (*The Japanese Oncology Journal*; 1137 words) and the longest (*The Journal of the American Medical Directors Association*, *The US Medicine Journal*; 31,609 words) journal guidelines (Table 1). Only 11 of the 54 guidelines examined contained keywords from all three non-neutral categories (Figure 1).

Table 3 shows the ratio of total (*per myriad*) numbers of keywords reflecting neutrality, welfare, the 3Rs and ARRIVE-acknowledgements to be approximately 2.5:1:1:1. Table 3 also reveals that the total number of references to regulatory bodies was greatest (53) at institutional level and least (10) for international guidelines.

**Table 3.** The sum of keywords categories (*per myriad*) reflecting neutrality, welfare, the 3Rs and ARRIVE-acknowledgements, and references to regulatory requirements (absolute count), found in 54 guidelines to authors representing seven medical specialty journals from nine countries. Two journals described adherence to the ‘Declaration of Helsinki’ as a regulatory requirement.

	Keyword categories				Regulatory level				
	Neutral	Welfare	3Rs	ARRIVE	institutional	National guidelines	Funding body	International guidelines	National law
Sum	465	184	201	197	53	26	16	10	16

**Figure 2.**
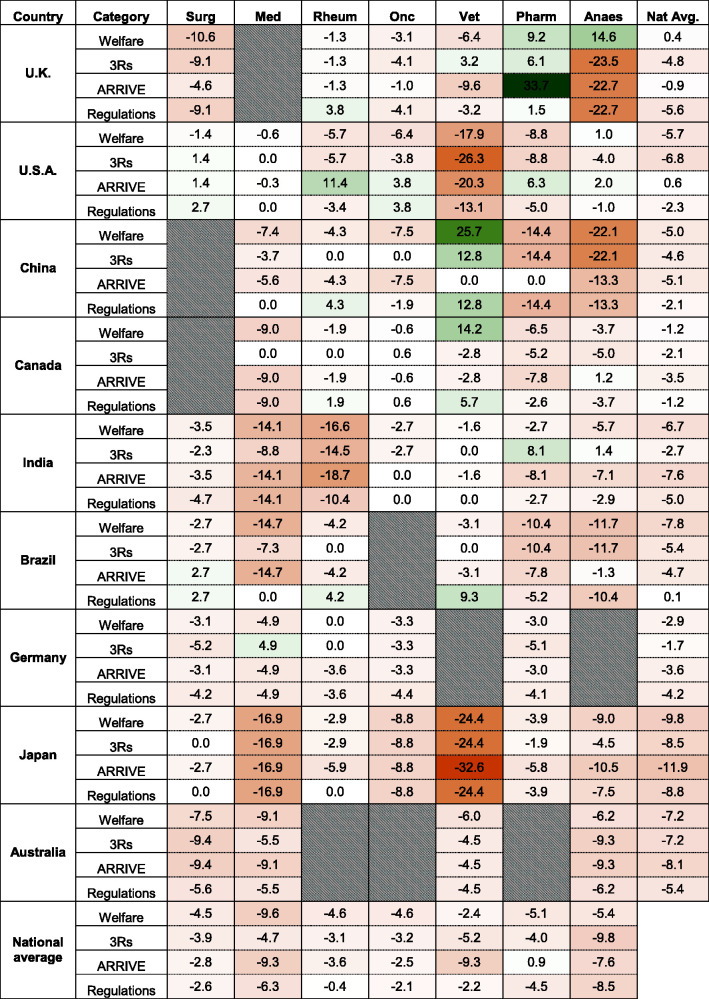
A ‘heat-map’ comparing non-neutral (welfare-, 3Rs and ARRIVE-related) and neutral keywords counts *per myriad* in GTAs from seven speciality journals from nine countries . Each integer equals the (non-neutral – neutral) keyword count divided *per myriad* (10,000) total word count. Grey hatched cells indicate national speciality journal guidelines that did not exist or failed to meet inclusion criteria. Negative integers in orange shaded cells indicates that neutral words exceeded welfare-related words. Green shaded cells indicate the reverse. The word count values and corresponding cell colours are:
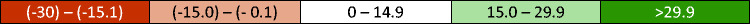

Figure 2 compares the non-neutral (welfare-, 3Rs- and ARRIVE-related) word counts with the number of neutral keywords counts *per myriad* by subtracting the latter from the former and colour-coding the result. The average (range) neutral word count was 8.6 *per myriad* (0.6–26.7) (Figure 1). Amongst the guidelines examined there was a significantly (*P* < 0.05) greater average non-neutral word count for veterinary journals when compared with medicine, oncology, rheumatology or surgery journals (Supplemental Figure S1a). *The British Journal of Anaesthesia* had the highest neutral word count at 26.7 *per myriad* whereas *The Journal of the American Medical Directors Association* had the lowest. A total of 16 journal guidelines made only one (neutral) reference to animal studies. These were “experiments” (10) “studies” (2) tests (2) “subjects” (1) and “science” (1). Single neutral references to animals were found in five of the eight rheumatology journal guidelines. British journals had significantly (*P* < 0.05) more non-neutral word counts than Australia, Canada, Germany and Japan. China also had more non-neutral word counts than Australia, Canada, Germany and Japan (*P* < 0.05) (Supplemental Figure S1b). In contrast, there were no significant differences (*P* > 0.05) between the non-neutral categories themselves: ‘welfare’, ‘3Rs’, ‘ARRIVE’ (*P* > 0.05; Supplemental Figure S1c).

The average (range) welfare-orientated keyword count was 3.4 *per myriad* (0–41.3), with *The British Journal of Anaesthesia* scoring the highest (Figure 1). Welfare-orientated keyword counts were completely absent in 24 of the 54 guidelines. All six Brazilian journal guidelines were devoid of welfare-oriented keywords. Only 5 of 54 journal guidelines (*The British Journal of Pharmacology*, *The British Journal of Anaesthesia*, *The Taiwanese Veterinary Journal*, *The Canadian Veterinary Journal* and *Anaesthesiology*) contained more welfare than neutral keywords (Figure 2).

The average (range) of 3Rs-orientated keyword count was 3.7 *per myriad* (0–19.3) (Figure 1). The highest 3Rs-related keyword counts were found in *The Veterinary Record* (UK) and *The Taiwanese Veterinary Journal*. A third of the guidelines contained no 3Rs-related keywords, including four out of the seven US journals (Figure 1). All Australian journals contained fewer 3Rs-related than neutral words. *The Journal of the American Veterinary Medical Association* had the lowest neutral to 3Rs-related score at –26.3 *per myriad*. On average, in all neutral to 3Rs-related scores across all 54 journals, there was a score of –5.09 *per myriad*. Only 8 of the 54 guidelines had more 3Rs-related than neutral keywords (Figure 2).

**Figure 3.**
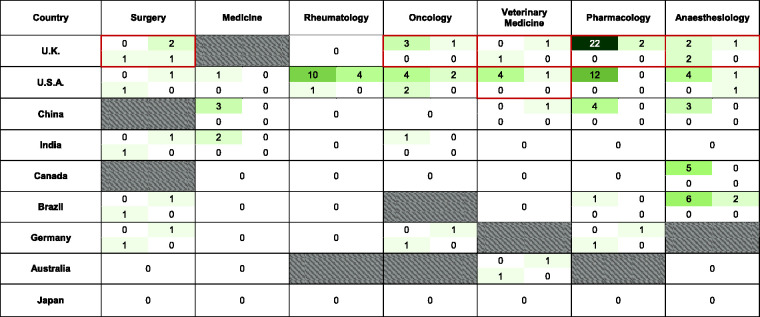
A ‘heat-map’ showing relative frequency of items referring to the ARRIVE guideline included in GTAs from seven speciality journals from nine countries. Within each country/speciality cell, the top left quadrant: ARRIVE-related keywords; top right quadrant: “ARRIVE”, bottom left quadrant: hyperlink to the ARRIVE guidelines; bottom right quadrant: citation for the ARRIVE guidelines. All word counts are absolute values. Grey hatched cells indicate national speciality journal guidelines that did not exist or failed to meet inclusion criteria. Red bordered cells indicate ARRIVE-endorsed journals (https://arriveguidelines.org/supporters/journals#B). The word count values and corresponding cell colours are:
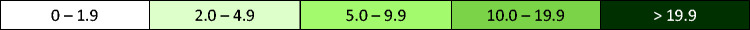

A total of 36 GTAs made no specific reference to the ARRIVE guidelines, either as an acronym, in the form of a URL (e.g. the NC3Rs website) or a citation to the original work (Figure 3). Of these, 27 contained no keywords associated with any ARRIVE requirements. The keyword ‘ARRIVE’ was absent from all seven Japanese journals, five of the six Canadian journals, three of the four Australian journals and seven of the eight rheumatology journals. The keyword was also missing from all eight medical, and seven of eight rheumatology guidelines. The average (range) of ARRIVE-orientated keyword count was 3.65 *per myriad* (0–36.7) (Figure 1). Only six of the selected Journals ‘endorse’ ARRIVE (Figure 3). *The British Journal of Pharmacology* scored the highest in ARRIVE keyword count *per myriad* at 36.7 0/000. Only eight guidelines had more ARRIVE-related words than neutral keywords in their GTAs (Figure 2). All Australian, Japanese, and German journals had fewer ARRIVE-related than neutral word counts.

**Figure 4.**
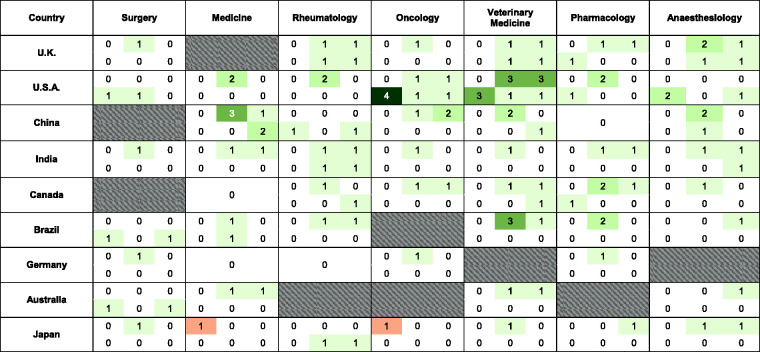
A ‘heat-map’ showing relative frequency of regulatory authorities required by GTAs from seven speciality journals from nine countries. Within each country/speciality cell, the top left sextant: “Declaration of Helsinki”; top mid sextant: Institutional welfare review board; top right sextant: National guidelines; bottom left sextant: funding body guidelines; bottom mid sextant: International guidelines; bottom right sextant: National law. Grey hatched cells indicate national speciality journal guidelines that do not exist or fail to meet inclusion criteria. The word count values and corresponding cell colours are:
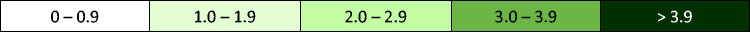

Four guidelines (*The Canadian Journal of Medicine*, *The German Medical Journal*, *The German Rheumatology Journal* and *The Chinese Journal of Pharmacology and Toxicology*) did not request the provision of any statements of approval (Figure 4). Two Japanese Journals (*The Japanese Journal of Cancer and Chemotherapy* and *Internal Medicine*) whilst referring to animal studies, requested authors to ‘refer to’ and ‘follow’ the Declaration of Helsinki 1964, which describes the ethical principles for medical research involving human subjects. A total of 15 guidelines required regulatory oversight by local institutional review bodies alone. Three of these were German journals and it seems pertinent that the remaining two German journals examined had no requirements to describe regulatory confinements. *The Journal of the American Veterinary Medical Association and Cancer* (US) required all five regulatory categories.

## Discussion

In attempting to quantify the importance placed on aspects of animal welfare required in submitted manuscripts, the GTAs of 54 journals were examined and the number of words associated with animal welfare, the 3Rs and ARRIVE compliance (non-neutral words) were compared with the word count for ‘animal’ in all other (neutral) contexts. The latter were also compared with the requirement for evidence of ethical and/or regulatory approval. In general, journal guidelines did not emphasise animal welfare, application of the 3Rs principles, or promote ARRIVE—and in equal measure. The mean keyword counts were similar in each of these categories and were approximately half the neutral word count (Table 3). Of the guidelines reviewed, 44% contained no keywords relating to animal welfare, whereas references to ARRIVE and the 3Rs were absent in 50% and 34%, respectively. The low emphasis placed on animal welfare and related topics in GTAs may explain the widespread contention that the description of welfare-related topics in published scientific material is inadequate.^3,17,28^

Previous studies examining specific aspects of welfare, e.g. analgesic provision,^5–8^ have unanimously concluded that there is room for significant improvement in the reporting of perioperative laboratory animal care. In the current study the words ‘pain’, ‘suffering’, ‘anaesthesia’ and ‘analgesia’ were categorised as welfare-related, which, of all the non-neutral categories, achieved the lowest word count (184) of all categories (Table 3). This suggests that the poor overall descriptions of animal welfare in scientific publications—a situation unaffected by the ARRIVE guidelines—may arise in part from the low emphasis the subject demands in GTAs.^10^

Welfare-related keywords were notably—and unaccountably—absent in all the Brazilian GTAs examined and in six of eight medical journals. Based on keyword count, the strongest requests for submitted articles to describe measures optimizing animal welfare were made—perhaps unsurprisingly—by veterinary medical and anaesthesia journals, with the greatest word count being found in *The British Journal of Anaesthesia* followed by *The Taiwanese Veterinary Journal*.

Lewis noted ‘the extent to which the 3Rs have been adopted and implemented by the scientific and medical research communities has been varied, both across continents and between research areas’,^29^ an opinion not entirely supported by the findings of the current study, in which 3Rs-related keyword counts exceeded those linked with welfare and the ARRIVE guidelines. While references to the 3Rs were absent in one-third of the GTAs examined, these were—except for the US—distributed evenly amongst the countries examined. Four guidelines of eight journals from the US unaccountably contained no keywords pertaining to the 3Rs. That the greatest 3Rs-related keyword counts were found in *The Veterinary Record* (UK) and *The Taiwanese Veterinary Journal* is also puzzling, as veterinary medicine is the one speciality in which animal replacement (at least) might prove counterproductive.

Both formal journal endorsement and a recommendation that an ARRIVE checklist be part of the editorial process have failed to improve compliance with the ARRIVE guidelines, leading to a suggestion that more stringent editorial policies are required.^10,14^ When the journal’s GTA of specific journal speciality (emergency medicine) or country of origin (mainland China) were examined,^12,22^ little (former) to no (latter) references to the ARRIVE guidelines were found. Poor endorsement of ARRIVE in a journal’s GTA was seen in the current study, although direct comparison between studies is not possible due to distinct inclusion methodologies. That the original (2010) ARRIVE guidelines have undergone recent revision indicates a failure to meet expectations and that ARRIVE uptake has been poor.^1–3,10,11,14^ The authors of the ARRIVE guidelines 2.0 acknowledge that ‘adherence to the [ARRIVE] guidelines has been inconsistent, and the anticipated improvements in the quality of reporting in animal research publications have not been achieved’.^3^ In the current study, the GTAs of all six ‘ARRIVE-endorsing’ journals examined (Figure 3) referred to ARRIVE directly, and in the form of an URL to the NC3Rs website, or in a citation to the original work. However, 36 GTAs referred to none of these. Furthermore, 27 of these 36 not only failed to acknowledge the existence of the ARRIVE guidelines but were devoid of any keywords that might have indicated implicit agreement with its objectives, i.e. ‘to maximise the output from research using animals by optimising the information that is provided in publications on the design, conduct, and analysis of the experiments’.^1^ The preponderance of ARRIVE-related keywords in GTAs of British and American journals, and their total absence from all Japanese guidelines would initially suggest a linguistic cause. However, moderate ARRIVE-related word counts found in Chinese, Indian, German and Brazilian GTAs undermines this possibility while accentuating the near absence of ARRIVE recognition in Australian and Canadian journals. A previous study found that a manuscript’s country of origin did not affect the level of ARRIVE compliance,^14^ which is at odds with the current findings. However, only PLOS ONE publications were analysed in that study, indicating that authors from different countries adhered to the same GTAs. The NC3Rs website^30^ (https://arriveguidelines.org/supporters/universities#Canada%20(Universities) shows ARRIVE endorsement by country and reveals a total absence of support from Indian, Brazilian and Japanese institutes, with single supporting institutes in China (Hong Kong) and Germany. This corresponds with the findings of the current study and provides a probable explanation.

Both the Committee on Publication Ethics (COPE) and leading publishers, e.g. Wiley, Elsevier, Sage and Springer require that authors identify the authority under which animal experiments are permitted. For example, COPE’s guidelines on good publication practice states that ‘Animal experiments require full compliance with local, national, ethical and regulatory principles, and local licensing arrangements’.^31^ That such mandates come from the publishers themselves possibly explains the high level of compliance found in almost all the GTAs examined in the current study, and in which the majority referred to Institutional Ethical Review Board approval. That said, four GTAs did not require any regulatory body identification, whilst two inexplicably referred to the ‘1964 Declaration of Helsinki’, which sets out ethical principles for medical research involving human subjects. At least two previous studies have examined factors affecting the citation of ethical review with one noting an improvement over the course of time,^10^ with the second recording a similar effect in Chinese articles possibly driven by the ARRIVE guidelines.^32^ It is impossible to determine if these factors were influential in the current study. It is possible that the existence of national regulations, and/or the diligence with which they are applied, might have affected the incidence of regulatory keywords in corresponding national GTAs. However, the animal welfare regulations in the nine countries studied differ markedly in complexity and application (and their description is beyond the scope of this study), which precludes drawing firm conclusions on this association.^21^

The inclusion/exclusion criteria used in the current study may have introduced bias. First, in analysing only GTAs, the emphasis that *The British Journal of Pharmacology* and *The British Journal of Cancer* place on animal welfare matters is understated considerably. The GTAs of both journals provide hyperlinks to supplemental articles containing extensive and explicit instructions on conducting and reporting animal experiments. For example, *The British Journal of Pharmacology* requires animal studies to adhere to its own guidelines, ARRIVE 2.0 and the supplement, *British Journal of Pharmacology: Updated guidance for 2020*. In this document, other additional requirements are referenced including *Transparency in Research involving Animals: The Basel Declaration and new principles for reporting research in BJP manuscripts*.^33^ Similarly, *The British Journal of Cancer*’s GTA references *Guidelines for the welfare and use of animals in cancer research*. Second, prioritising practicality over the inclusion/exclusion criteria used may have led to unrepresentative journals being selected. For example, only journals with online GTAs that publish animal studies (based on the word ‘animal(s)’ appearing at least once in the GTA) were analysed. For this reason, *The Canadian Journal of Surgery*’s guidelines were excluded even though it publishes animal research. Third, restricting analyses to English language versions may also have been prejudicial: the use of translational software, as in a previous study,^22^ would have allowed the inclusion of two, arguably more appropriate, German journals, i.e. *Deutsche Tierarztliche Wochenschrift* and *Anasthesiologie und Intensivmedizin*. However, automated translation software such as Google Translate might have changed meanings. Fourth, restricting analyses to the GTA alone may have excluded journals in which selected keywords existed elsewhere on the journal’s website, for example, under ‘Editorial Policies’. Fifth, suitable journals may have been excluded because examination was limited to electronic guidelines, although this has been common practice in studies involving GTA analysis.^12,22,34,35^

Finally, difficulty in identifying ‘national’ journals may have led to considerable mis-selection. For example, the exclusion of international journals—identified through self-proclamation and the multi-national distribution of editorial offices—meant several high profile (and presumably influential) journals were not represented in the current study. The exclusion of regional journals may have prevented Australian and Japanese journals receiving fair representation as all journals excluded for this reason were from the Asia-Pacific region. Defining Chinese journals was problematic insofar that previous (Chinese) authors conducting analyses of Chinese scientific output have disregarded work originating in Hong Kong, Macau and Taiwan.^12^ In the current study, *The Taiwanese Veterinary Journal* and *The Asian Journal of Anesthesiology* were regarded as Chinese because equivalent journals published in the People’s Republic of China failed to meet other inclusion criteria.

In previous work, keywords have been categorised by several authors examining text independently or by a single examiner.^12,22^ In the current study, only two of the three authors analysed a portion of the selected GTAs for keyword categorisation and so an opportunity to examine inter-observer variation was lost. However, the iterative process by which keywords were initially re-categorised until agreement was reached was successful, although time-consuming.

Given the methodological problems identified previously, a case may be made for repeating the current study with improved inclusion/exclusion criteria. However, knowing more confidently whether national origins and/or medical speciality affects the emphasis placed on animal welfare in scientific publications would reveal only whether and where remedial action was required; it would not guarantee that remedial action would be effective. Nevertheless, it is proposed that future research focus on developing more efficient methods of GTA analysis using advanced keyword recognition techniques, thus allowing larger datasets to be established more rapidly.

In conclusion, this study has identified that, in general, the GTAs of most journals do not emphasise the need to record details affecting animal welfare, and, subsequently, data quality, in submitted journals. Methodological limitations preclude attempts to identify differences attributable to nationality and/or medical speciality in attitudes to laboratory animal welfare.

## Supplemental Material

sj-pdf-1-lan-10.1177_00236772221097825 - Supplemental material for Animal welfare requirements in publishing guidelines Click here for additional data file. Supplemental material, sj-pdf-1-lan-10.1177_00236772221097825 for Animal welfare requirements in publishing guidelines by Amanda L Novak, Darren J. Shaw and R. Eddie Clutton in Laboratory Animals
