# Supplementary figures and images for: Animal welfare requirements in publishing guidelines
Source: Lab Anim. 2022 Jun 21;56(6):561–75. doi: 10.1177/00236772221097825 (PMC9709535; doi:10.1177/00236772221097825)

(a) Journal subject differences

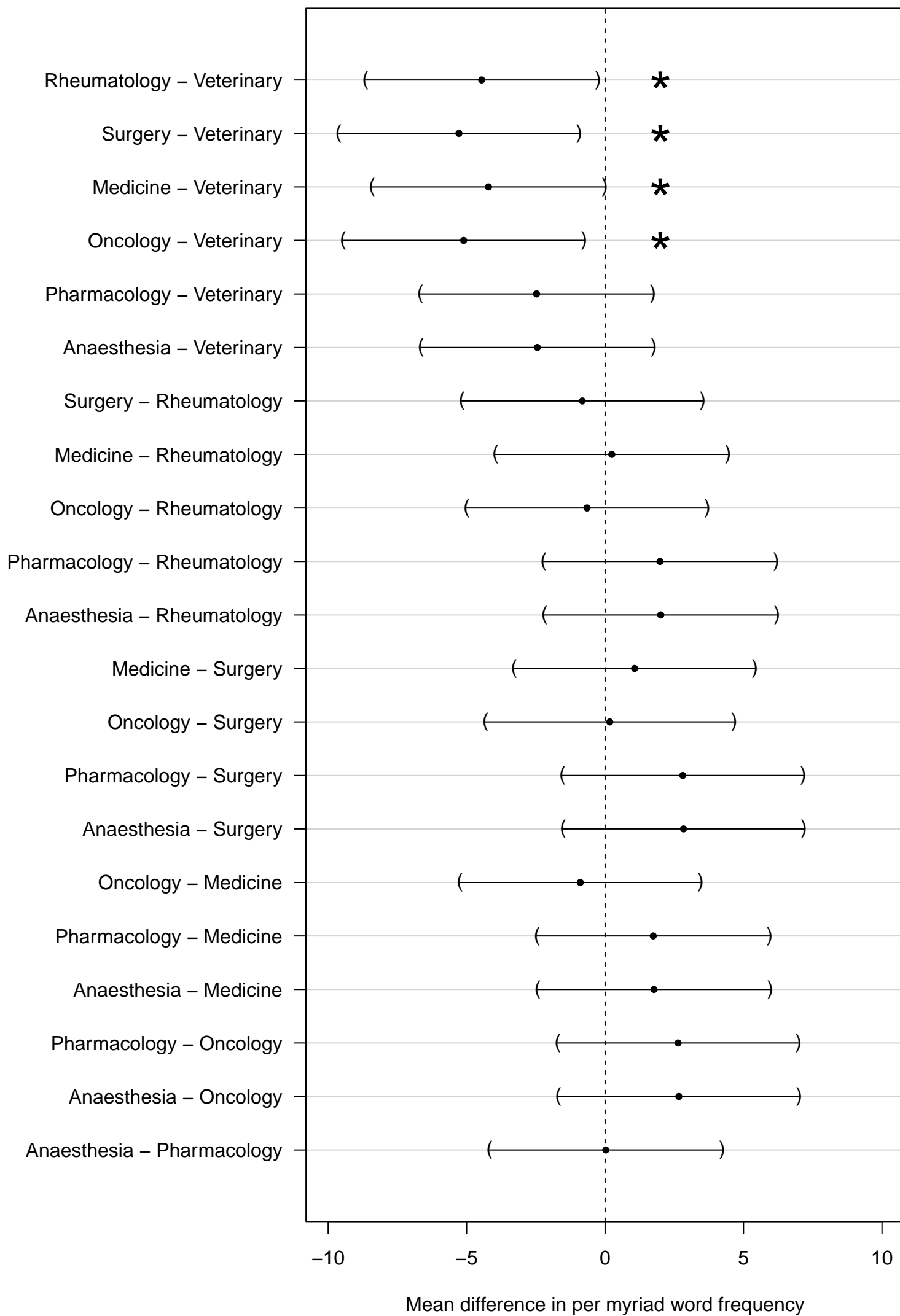

## (b) Country differences

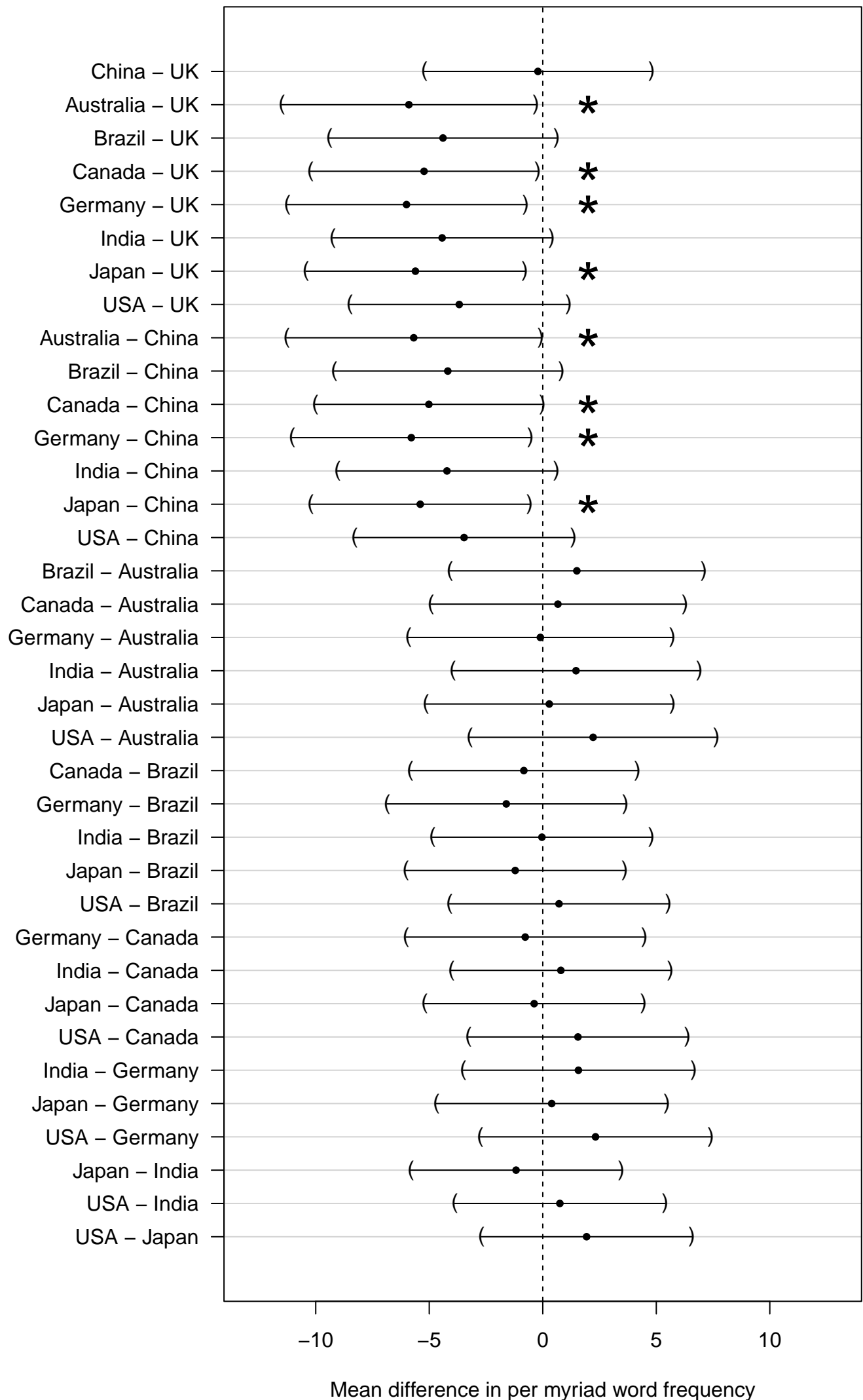

(c) Terms of interest differences

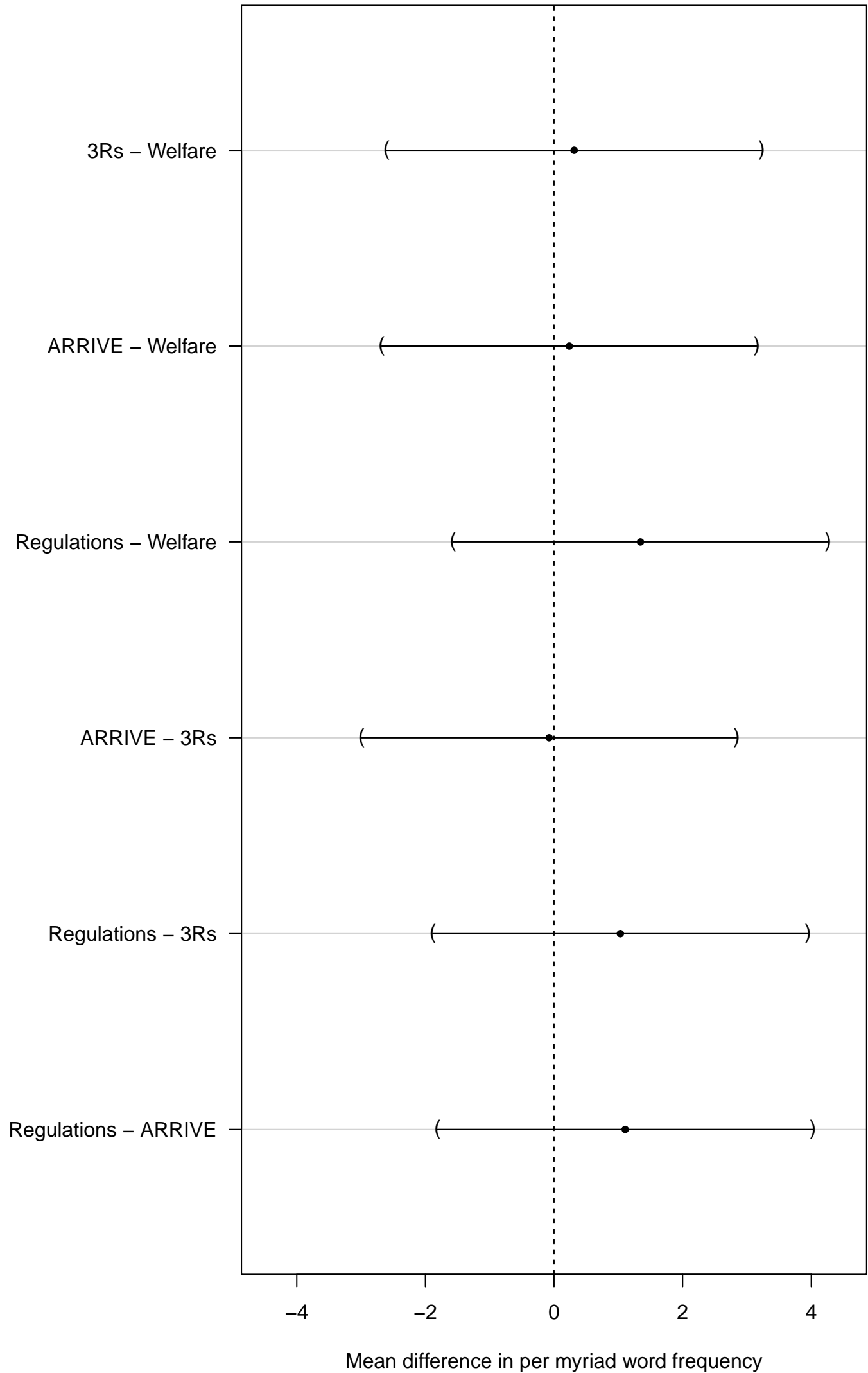

Supplement: sj-pdf-1-lan-10.1177_00236772221097825 - Supplemental material for Animal welfare requirements in publishing guidelines [file sj-pdf-1-lan-10.1177_00236772221097825.pdf]
